# Pre-incubation with hucMSC-exosomes prevents cisplatin-induced nephrotoxicity by activating autophagy

**DOI:** 10.1186/s13287-016-0463-4

**Published:** 2017-04-08

**Authors:** Bingying Wang, Haoyuan Jia, Bin Zhang, Juanjuan Wang, Cheng Ji, Xueming Zhu, Yongmin Yan, Lei Yin, Jing Yu, Hui Qian, Wenrong Xu

**Affiliations:** 1 Key Laboratory of Laboratory Medicine of Jiangsu Province, School of Medicine, Jiangsu University, 301 Xuefu Road, Zhenjiang, Jiangsu 212013 People’s Republic of China; 2 The Second Affiliated Hospital of Soochow University, Suzhou, Jiangsu People’s Republic of China

**Keywords:** Human umbilical cord mesenchymal stem cell, Exosome, Cisplatin, Nephrotoxicity, Autophagy

## Abstract

**Background:**

The administration of cisplatin is limited due to its nephrotoxic side effects, and prevention of this nephrotoxicity of cisplatin is difficult. Mesenchymal stem cell (MSC)-derived exosomes have been implicated as a novel therapeutic approach for tissue injury. In this study, we demonstrated that the pretreatment of human umbilical cord MSC-derived exosomes (hucMSC-Ex) can prevent the development of cisplatin-induced renal toxicity by activation of autophagy in vitro and in vivo.

**Methods:**

In vitro, rat renal tubular epithelial (NRK-52E) cells were pre-incubated with exosomes from hucMSC or HFL1 (human lung fibroblast cells; as control) for 30 min, and 3-methyladenine (an autophagic inhibitor) and rapamycin (an autophagic inducer) for 1 h before cisplatin treatment for 8 h, respectively. Cells were harvested for apoptosis assay, enzyme-linked immunosorbent assay (ELISA), Western blot, and quantitative real-time polymerase chain reaction (qRT-PCR). In vivo*,* we constructed cisplatin-induced acute kidney injury rat models. Prior to treatment with cisplatin for 0.5 h, hucMSC-Ex or HFL1-Ex were injected into the kidneys via the renal capsule. 3-methyladenine and rapamycin were injected under the kidney capsule before hucMSC-Ex. All animals were sacrificed at 3 days after cisplatin injection. Renal function, Luminex assay, tubular apoptosis and proliferation, and autophagy response were evaluated.

**Results:**

hucMSC-Ex inhibited cisplatin-induced mitochondrial apoptosis and secretion of inflammatory cytokines in renal tubular epithelial cells in vitro. hucMSC-Ex increased the expression of the autophagic marker protein LC3B and the autophagy-related genes ATG5 and ATG7 in NRK-52E cells. Rapamycin mimicked the effects of hucMSC-Ex in protecting against cisplatin-induced renal injury, while the effects were abrogated by the autophagy inhibitor 3-methyladenine in the animals.

**Conclusions:**

Our findings indicate that the activation of autophagy induced by hucMSC-Ex can effectively relieve the nephrotoxicity of cisplatin. Therefore, pre-treatment of hucMSC-Ex may be a new method to improve the therapeutic effect of cisplatin.

**Electronic supplementary material:**

The online version of this article (doi:10.1186/s13287-016-0463-4) contains supplementary material, which is available to authorized users.

## Background

Cisplatin is a platinum inorganic complex, extensively used in clinical chemotherapy for many solid tumors. Administration of a high dose of cisplatin is limited due to its nephrotoxic side effects [1, 2]. Therefore, preventive measures are needed to counteract the renal damage induced by cisplatin. It is now recognized that exosomes/microvesicles as nanoparticles are an integral part of the intercellular microenvironment [3], and may play a pivotal role in cell-to-cell communication [4]. Microvesicles (MVs) are circular fragments of membrane released from the endosomal compartment as exosomes or shed from the surface membranes of most cell types. Exosomes/microvesicles may interact with target cells by surface-expressed ligands and transfer surface receptors, and deliver proteins, mRNA, and bioactive lipids between cells [5, 6]. Recently, many studies have revealed that mesenchymal stem cell (MSC)-derived exosomes or microvesicles can promote tissue regeneration. MVs from human bone marrow-derived MSCs contribute to favor functional and morphological recovery in rodent models of acute kidney injury (AKI) induced by glycerol [7], ischemia/reperfusion [8], and cisplatin [9]. The research implies that exosomes from MSCs may be a novel stem cell-based therapy for kidney diseases. Our previous works indicate that human umbilical cord-derived MSCs (hucMSCs) improve the recovery of ischemia/reperfusion-induced rat renal injury via anti-apoptotic and anti-inflammatory mechanisms [10–12]. We also demonstrated that hucMSC-derived exosomes (hucMSC-Ex) protect against cisplatin-induced renal oxidative stress and apoptosis, alleviate CCl_4_-induced liver fibrosis, and enhance cutaneous wound healing [13–17]. All the above studies show that hucMSC-Ex can improve the tissue injury. However, it is unknown whether hucMSC-Ex administration before injury can prevent kidney damage in the early stages.

Studies have demonstrated that autophagy is critical for normal proximal tubule function and protection against acute tubular injury [18, 19]. There is a dynamic feedback between autophagy and cellular energy balance [20]. Autophagy not only plays a principal role in the supply of nutrients for cell survival, but also plays an active role in cellular homeostasis, where it acts as a cytoplasmic quality-control regulator to eliminate long-lived or unfolded proteins and damaged organelles [21, 22]. These reports revealed that the activation of autophagy plays an important role in relieving tissue damage. Whether hucMSC-Ex can promote autophagy activation is unclear.

Herein, we show that hucMSC-Ex had a markedly preventative effect against cisplatin-induced renal toxicity, and we explored the mechanism of action. Our findings indicate that hucMSC-Ex promoted autophagy in renal tubule epithelial cells and kidney tissues by cisplatin induced through the inhibition of mTOR, thus alleviating cell apoptosis and inflammatory response in the early stages.

## Methods

The study was approved by the ethical committee of Jiangsu University (2012258).

### Isolation and culture of hucMSCs

After obtaining parental and ethics committee consent, the fresh umbilical cords were collected and processed within 6 h as described previously [23]. Umbilical cords were cut into pieces and floated in low glucose Dulbecco’s modified Eagle’s medium (LG-DMEM) containing 10% fetal bovine serum (FBS; ExCell Biology, China) and 1% penicillin and streptomycin. Cord pieces were subsequently incubated at 37 °C in humid air with 5% CO_2_. When well-developed colonies of fibroblast-like cells reached 80% confluency, cultures were trypsinized into new flasks for further expansion. hucMSCs were identified by fluorescence-activated cell sorting (FACS) and differentiation experiments.

### FACS analysis and differentiation studies of hucMSCs

hucMSCs were resuspended in phosphate-buffered saline (PBS). Cell aliquots (300 μl) were incubated with fluorescein isothiocyanate (FITC)-conjugated or phycoerythrin (PE)-conjugated monoclonal antibodies specific for CD13, CD29, CD44, CD90, CD105, HLA-1, CD45, HLA-DR, and CD34 on ice for 30 min. FITC or PE mouse nonimmune isotypic IgG were used as controls. All antibodies were purchased from BD Company. The pluripotency of hucMSCs was confirmed by their ability to differentiate into osteocytes and adipocytes, as described previously [23].

### Isolation and characterization of exosomes from hucMSCs and HFL1 cells

As control cells, human fetal lung fibroblast-1 (HFL1) cells were obtained from Cell Bank, Type Culture Collection Committee, the Chinese Academy of Sciences (Shanghai, China). In order to allow exosome separation, HFL1 cells were cultured with serum-free α-modified minimum essential medium (α-MEM); hucMSCs were cultured with serum-free LG-DMEM. The conditioned medium was collected at 48 h. Density gradient centrifugations were executed and the procedure for the extraction and purification of exosomes was performed as described previously [13, 14]. The purified exosomes were stored at –86 °C until use. The expression of the exosomal makers CD81, CD9, and CD63 was detected using Western blot. The purified exosomes were further identified by nanoparticle tracking analysis (NTA) system (Version 2.3 Build 0006 BETA2) and transmission electron microscopy.

### NTA and transmission electron microscopy

In order to analyze the characteristics of the exosomes, particle size, substantial shape, and the relative-intensity three-dimensional plot of hucMSC-Ex were tested using the NTA system. The purified hucMSC-Ex were applied to glow-discharged carbon-coated copper grids to detect the precise shape. PTA (phosphor-tungsten acid) staining was conducted for 30 min. The grids were then rinsed with droplets of deionized water and dried. Ultra-thin sections of NRK-52E cells were prepared to observe the formation of autophagic bodies. Transmission electron micrographs were recorded using a Tecnai 12 (Philips, Holland).

### In vitro experiment

Normal rat kidney epithelial (NRK-52E) cells were obtained from Cell Bank (Shanghai, China). NRK-52E cells were pre-incubated with exosomes from hucMSC (200 μg/ml) or exosomes from HFL1 (200 μg/ml) for 30 min, followed by treatment with cisplatin (8 μM) for 8 h. 3-methyladenine (3MA; 1 mg/ml; Sigma, USA) or rapamycin (200 μg/ml; Sigma) was given for 0.5 h before exosomes treatment and 1 h before cisplatin treatment in NRK-52E cells, respectively.

### In vivo rat model

Female Sprague-Dawley rats at 6–8 weeks old, weighing 210–250 g, were used. The animals were kept under standard laboratory conditions (12 h light/12 h dark cycle, and 21 ± 2 °C). Animals were divided into seven groups of six rats each and treated as follows: 1) control group (no cisplatin treatment); 2) PBS group (intraperitoneal injection of a single dose of 5 mg/kg cisplatin); 3) hucMSC-Ex group (0.5 h before cisplatin administration both kidneys in one rat received a renal capsule injection of 200 μg hucMSC-Ex); 4) HFL1-Ex group (0.5 h before cisplatin administration both kidneys in one rat received a renal capsule injection of 200 μg HFL1-Ex); 5) hucMSC-Ex + 3MA group (500 μg/kidney 3MA was injected under the kidney capsule before hucMSC-Ex and cisplatin administration); 6) 3MA group (500 μg/kidney 3MA was injected under the kidney capsule before cisplatin administration); and 7) Rapa group (20 μg/kidney 3MA was injected under the kidney capsule before cisplatin administration).

All animals were sacrificed at 3 days after cisplatin injection. Kidney tissue specimens were divided into two. One was quickly excised, rinsed in ice-cold saline, and fixed in 4% paraformaldehyde, and the paraffin-embedded tissues were sliced and stained with hematoxylin and eosin (H&E). Sections were analyzed and at least 20 random fields were scored by a nephropathologist in a blinded manner. Tubular cell necrosis, tubular dilation, and tubular protein casts (200× magnification) in sections were observed and analyzed. Abnormalities were graded by a semiquantitative score from 0 to 4: 0, no abnormalities; 1, changes affecting less than 25% of the sample; 2, changes affecting 25 to 50%; 3, changes affecting 50 to 75%; 4, changes affecting more than 75%. The other specimen was used immediately or frozen at –86 °C until further biochemical analysis. Blood samples were collected before and after cisplatin injection (0, 1, 2, and 3 days). Serum creatinine (Cr) and blood urea nitrogen (BUN) levels were measured by an automatic biochemical analyzer (AU2700; Olympus). Serum levels of inflammatory cytokines such as tumor necrosis factor (TNF)-α, interleukin (IL)-1β and IL6 were determined by Luminex assays (Luminex 200; Millipore).

### Mitochondrial membrane potential assay

NRK-52E cells seeded in 24-well plates were treated as described for the in vitro experiment. After treatment with cisplatin for 8 h, the cells were fixed with 4% paraformaldehyde. Mitochondrial membrane potential was measured by a JC-1 kit according to the manufacturer’s protocol (Beyotime, China).

### RNA extraction and quantitative real-time PCR

Total RNA was extracted with Trizol reagent (Invitrogen, USA) from NRK-52E cells and kidney tissues; cDNA was synthesized using a reverse transcription kit according to the manufacturer’s instructions (Vazyme, China). Quantitative real-time polymerase chain reaction (qRT-PCR) was used to detect the expression of ATG5, ATG7, and β-actin genes. All samples were examined in triplicate, and all reactions were repeated three times independently using the CFX96 Touch™ Real-Time PCR Detection System (Bio-Rad, USA).

### TUNEL assay

Terminal deoxynucleotidyl transferase-mediated dUTP nick end-labeling (TUNEL) staining was used to detect the apoptotic cells. hucMSCs, HFL1 cells, NRK-52E cells, and the kidney tissues were examined using the TdT-FragEL DNA fragmentation detection kit according to the manufacturer's protocol (Boster, China). Positive cells were identified as dark brown nuclei under a light microscope (Nikon eclipse TE 3000-U, Japan). The number of apoptotic cells was counted in 20 randomly selected visual fields of blinded samples, at 200× magnification.

### Western blot

Cells, exosomes, and tissues were lysed in RIPA buffer. Protein concentration was determined using the BCA assay kit (Pierce, USA). Sources and dilution factors of primary antibodies were: rabbit polyclonal CD63 (1:1000; Bioworld, USA), CD9 (1:1000; Bioworld), CD81 (1:1000; Epitomics, USA), PCNA (1:1000; Bioworld), BCL-XL (1:100; SAB, USA), BCL-2 (1:1000; Bioworld), Bax (1:1000; Bioworld), Cytochrome C (1:500; Abcam, USA), caspase-3 (1:500; Bioworld), IL-1β (1:500; Bioworld,), LC3B (1:500; Abcam), Beclin-1 (1:600; Proteintech, USA), mTOR (1:500; SAB), p-mTOR (1:500; SAB), 4EBP1 (1:200; SAB), p70S6K (1:200; SAB), and mouse monoclonal GAPDH (1:3000; Kang Chen, China). The nucleoprotein and plasma protein was separated by the nuclear and plasma protein isolation kit (Vazyme, China), with the primary antibody NF-kB-P65 in the nucleus (1:500; SAB), primary antibody nucleoprotein Histone (1:1000; SAB). After incubation with the primary antibodies overnight at 4 °C, membranes were washed three times with Tris-buffered saline with 0.05% Tween-20 and challenged with HRP-conjugated goat anti-rabbit or goat anti-mouse antibody (1:2000; Bioworld). Western blot was performed by Luminata™ crescendo western HRP substrate (Millipore, USA) and analyzed using MD Image Quant Software.

### Immunocytochemistry and immunohistochemistry

NRK-52E cells, treated as described for the in vitro experiment, were fixed in 4% paraformaldehyde and permeabilized with HEPES-Triton X100 buffer. The paraffin-embedded kidney tissue sections were dewaxed. Endogenous peroxidase activity was then inhibited by exposure to 3% hydrogen peroxide for 10 min. Subsequently, the sections were boiled for 10 min in citrate buffer (pH 6.0, 10 mM) for antigen retrieval. The sections were then blocked with 5% BSA (Boster Bioengineering, Co. Ltd., Wuhan, China) and incubated with TNF-α (1:100; Bioworld) and PCNA (1:100; Bioworld) primary antibody at 37 °C for 1 h. After the sections were washed with PBS, they were then incubated with diluted secondary antibody for 20 min. Finally, cells were visualized using diaminobenzidine (DAB) substrate and counterstained with hematoxylin for microscopic examination. The number of PCNA-positive cells were counted in 20 randomly selected visual fields of blinded samples, at 200× magnification.

### Enzyme-linked immunosorbent assay

NRK-52E cells were treated as mentioned above. Culture supernatants were collected and cell debris was removed by centrifugation. Supernatants were examined using the rat TNF-α enzyme-linked immunosorbent assay (ELISA) kit according to the manufacturer's protocol (ExCell Biology, China).

### Luminex assay

Female Sprague-Dawley rats were treated as mentioned above. The serum inflammatory cytokines TNF-α, IL-1β, and IL6 were measured using Luminex kits (Millipore, Billerica, MA, USA) according to the manufacturer’s instructions.

### Cell transfection and structured illumination microscopy assay

NRK-52E cells were seeded in 24-well plates and cultured for 24 h, then transfected with mRFP-GFP-LC3 adenovirus according to the manufacturer's protocol (Han Heng Biology, China). Then, cells were cultured on coverslips and divided into four groups: control; PBS; pre-treated hucMSC-Ex; and pre-treated HFL1-Ex. After treatment, the cells were washed with PBS, fixed with 4% paraformaldehyde, and permeabilized with HEPES-Triton X100 buffer. Finally, the cells were stained with Hoechst33342 for nuclear staining, and the images were acquired with a structured illumination microscopy (Nikon, SIM).

### Statistical analysis

All data from different experiments are expressed as mean ± SD. Statistical analysis was performed by Student’s *t* test or by analysis of variance (ANOVA) with Newmann-Keuls multicomparison or Dunnett’s post hoc tests as appropriate. A two-tailed *P* value <0.05 was considered statistically significant.

## Results

### Characterization of hucMSC and hucMSC-Ex

MSCs isolated from the umbilical cord were characterized by FACS analysis (see Additional file 1: Figure S1A) and induced differentiation (Additional file 1: Figure S1B and C). Exosomes were extracted and purified from hucMSC and were identified by morphology and exosomal markers. The particle size and concentration of exosomes were measured by an NTA system, peaking at 102 nm diameter (see Additional file 2: Figure S2Aa), a relative-intensity three-dimensional plot (shown in Additional file 2: Figure S2Ab), and electron micrograph of phosphotungstanic acid-stained exosomes (Additional file 2: Figure S2Ac). The results of transmission electron microscopy showed that the hucMSC-Ex had a fingerprint-like membrane structure (Additional file 2: Figure S2Ba) and a spheroid shape (Additional file 2: Figure S2Bb). The purified hucMSC-Ex expressed exosomal marker proteins such as CD81, CD9, and CD63 (Additional file 2: Figure S2C). TUNEL stain showed that the number of apoptotic cells was not significantly different between normal cultured conditions and serum-free cultured conditions at 48 h (Additional file 2: Figure S2D). Electron microscope examination showed that hucMSC-Ex expressed CD9 colloidal gold (Additional file 2: Figure S2E).

### hucMSC-Ex can prevent cisplatin-induced AKI in vivo

**Fig. 1:**
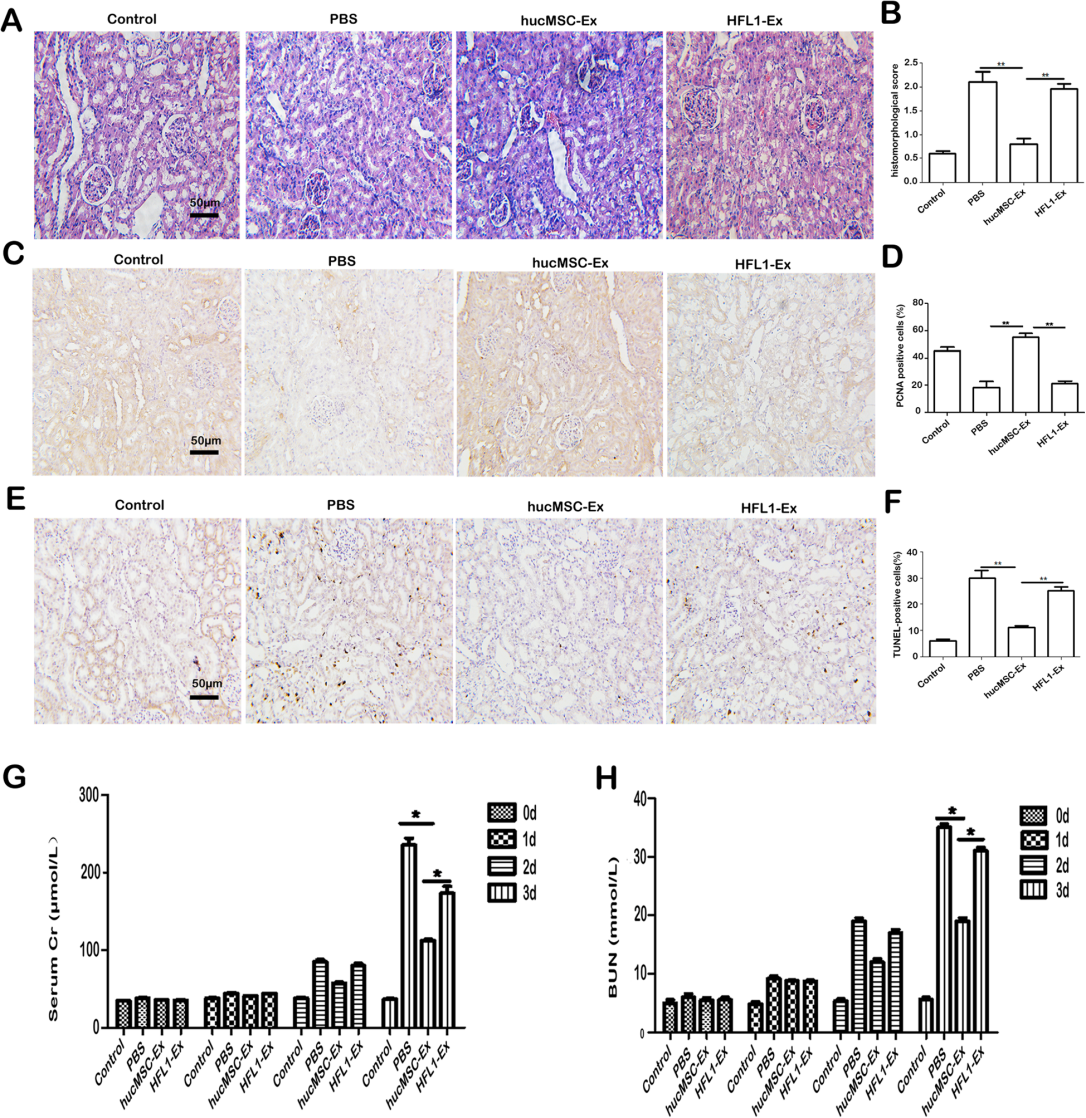
Human umbilical cord-derived mesenchymal stem cell exosomes (*hucMSC-Ex*) prevent deterioration of renal function in vivo. **a** Representative images of renal histology (200×, *scale bar* = 50 μm). **b** The histomorphological score. **c** Immunohistochemical analysis of PCNA expression in kidney tissues (200×, *scale bar* = 50 μm). **d** Percentage of proliferating cell nuclear antigen (*PCNA*)-positive cells. **e** TUNEL assay for apoptotic cells in rat renal tissues (200×, *scale bar* = 50 μm). **f** Percentage of apoptotic cells. Serum creatinine (*Cr*) (**g**) and blood urea nitrogen (*BUN*) (**h**) levels were measured at different times after cisplatin injection. Data are expressed as mean ± SD. *P < 0.05, *n* = 6. Rats treated as mentioned for the in vivo rat model were divided into four groups as follows: 1) control; 2) phosphate-buffered saline (*PBS*); 3) hucMSC-Ex; and 4) human fetal lung fibroblast-1exosomes (*HFL1-Ex*). All animals were sacrificed at 3 days after cisplatin injection

We determined whether hucMSC-Ex could prevent AKI in rats. Rats were divided into four groups (*n* = 6 per group) as follows: 1) control group (rats were treated with PBS intraperitoneally); 2) PBS group (rats were injected with intraperitoneal cisplatin at 5 mg/kg body weight; PBS was injected under the kidney capsule before treatment with cisplatin); 3) hucMSC-Ex group (hucMSC-Ex (200 μg/kidney) were injected under bilateral renal capsules before treatment with cisplatin); and 4) HFL1-Ex group (HFL1-Ex (exosomes derived from HFL1, as a control for hucMSC-Ex; 200 μg/kidney) was injected under the kidney capsule before treatment with cisplatin. All animals were sacrificed at 3 days after cisplatin injection. Representative images of H&E staining showed that the number of renal tubules with edema and structural damage were significantly reduced in the hucMSC-Ex group (Fig. 1a and b). The number of PCNA-positive cells was higher in hucMSC-Ex group compared to the PBS and HFL1-Ex groups (Fig. 1c and d). In contrast, the number of TUNEL-positive cells was lower in the hucMSC-Ex group compared to the PBS and HFL1-Ex groups (Fig. 1e and f). We also measured the serum creatinine (Cr) and blood urea nitrogen (BUN) levels at different times after cisplatin injection. We found that cisplatin led to a sharp increase in both Cr and BUN levels at 3 days after injection. Treatment with hucMSC-Ex significantly decreased Cr and BUN levels (Fig. 1g and h). In summary, these results indicate that hucMSC-Ex protects against renal injury induced by cisplatin, but there is no similar preventive action in the HFL1-Ex group.

### hucMSC-Ex inhibits cisplatin-induced apoptosis and inflammatory response in vitro

**Fig. 2:**
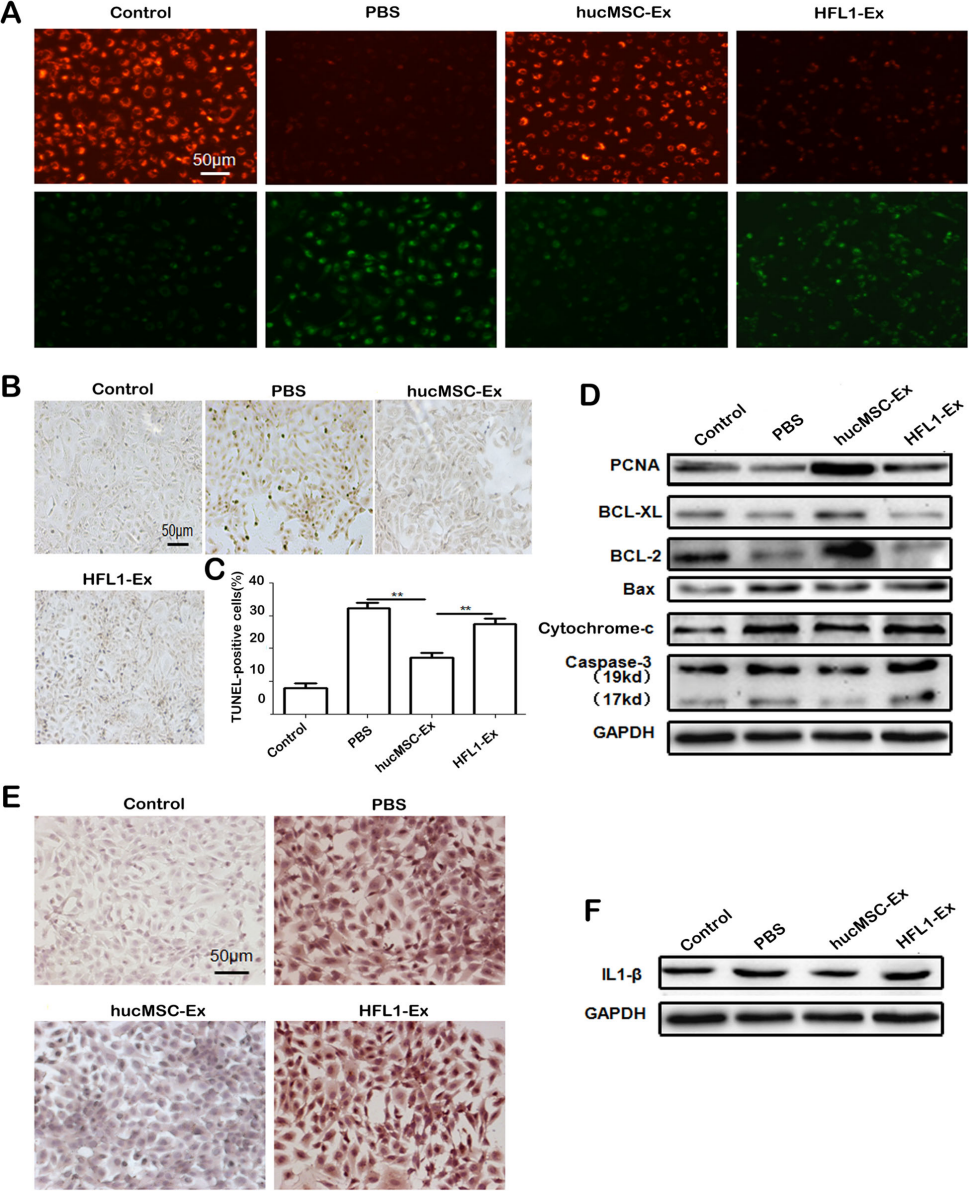
Human umbilical cord-derived mesenchymal stem cell exosomes (*hucMSC-Ex*) inhibit cisplatin-induced apoptosis and secretion of inflammatory cytokines in NRK-52E cells in vitro. **a** NRK-52E cells were stained with JC-1 dye and observed under fluorescent microscopy (JC-1 produces *red* fluorescence within the mitochondria as JC-1-aggregates while it emits *green* fluorescence when it leaks into the cytoplasm as JC-1-monomers; the shift between *green* and *red* is proportional to the mitochondrial membrane potential change) (200×, *scale bar* = 50 μm). **b** TUNEL assay was performed to detect the apoptotic cells (200×, *scale bar* = 50 μm). **c** Percentage of apoptotic cells. **P < 0.01, *n* = 3. **d** The expression of proliferating cell nuclear antigen (*PCNA*), BCL-XL, BCL-2, Bax, Cytochrome-c, cleaved Caspase-3, and GAPDH proteins was evaluated by Western blot. **e** Immunohistochemical analysis of tumor necrosis factor alpha (*TNF-α*) expression (200×, *scale bar* = 50 μm). **f** The expression of interleukin 1-β (*IL1-β*) and GAPDH proteins was evaluated by Western blot. NRK-52E cells treated as mentioned for the in vitro experiment were divided into four groups: 1) control; 2) phosphate-buffered saline (*PBS*); 3) hucMSC-Ex; and 4) human fetal lung fibroblast-1exosomes (*HFL1-Ex*)

To determine whether hucMSC-Ex prevents renal tubule epithelial cells from cisplatin-induced apoptosis and secretion of inflammatory cytokines, NRK-52E cells (rat kidney epithelial cells) were treated as described for the in vitro experiment. Mitochondrial membrane potential is considered a sensitive measurement of early apoptosis. Compared with the control group (NRK-52E cells without cisplatin), cisplatin reduced the mitochondrial membrane potential. hucMSC-Ex, but not HFL1-Ex, treatment antagonized the cisplatin-induced decrease in mitochondrial membrane potential (Fig. 2a). We also confirmed the effect of hucMSC-Ex on cisplatin-induced apoptosis using the TUNEL assay. As shown in Fig. 2b and c, the number of TUNEL-positive cells, identified as dark brown nuclei, was lower in the hucMSC-Ex group than that in the PBS and HFL1-Ex groups. After treatment with hucMSC-Ex, the expression of apoptosis-related proteins such as Bax, Cytochrome c, and cleaved Caspase-3 decreased, but the expression of proliferative and anti-apoptotic proteins such as PCNA, BCL-XL, and BCL-2 increased (Fig. 2d). To determine whether hucMSC-Ex affects the cisplatin-induced inflammatory response in renal tubule epithelial cells, we detected the expression of TNF-α by immunohistochemical staining and IL1-β by Western blot. The results showed that the levels of TNF-α (Fig. 2e) and IL1-β (Fig. 2f) were reduced in the hucMSC-Ex group compared to that in the PBS group. However, HFL1-Ex showed minimal effects on TNF-α and IL1-β expression. Taken together, these results suggest that hucMSC-Ex can inhibit cisplatin-induced apoptosis and secretion of inflammatory cytokines in renal tubule epithelial cells.

### hucMSC-Ex activated autophagy in vitro

**Fig. 3:**
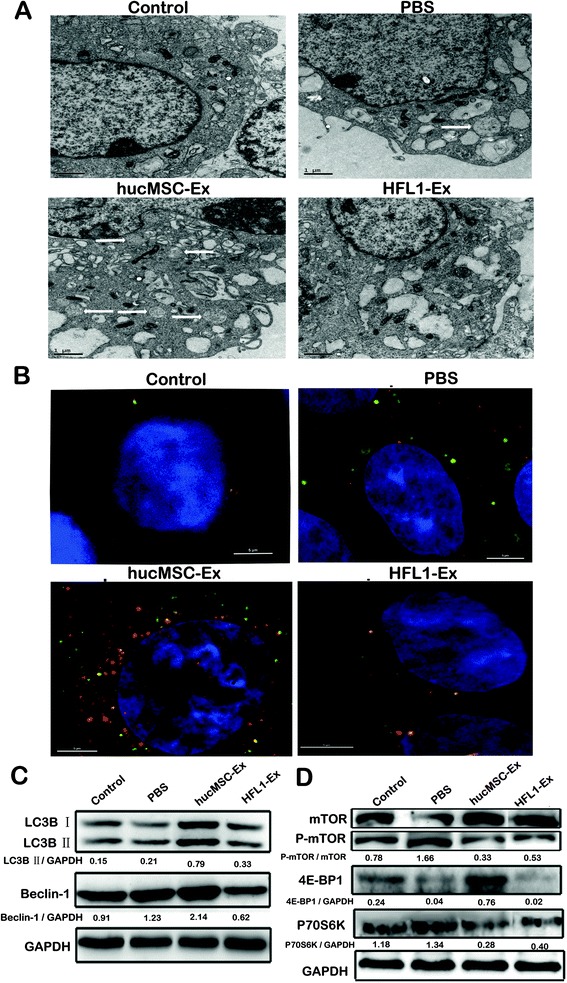
Human umbilical cord-derived mesenchymal stem cell exosomes (*hucMSC-Ex*) induce autophagy in NRK-52E cells in response to cisplatin. **a** Transmission electron microscopic examination of autophagosomes in NRK-52E cells. The representative images indicate typical autophagosomes in the cells treated with hucMSC-Ex (*scale bar* = 1 μm). **b** SIM examination of autophagosomes in NRK-52E cells. *Yellow dots* represents autophagosomes, *red dots* represent autolysosomes (*scale bar* = 5 μm). Representative images of fluorescent LC3 puncta are shown. **c** Western blotting analyses of the expression of the autophagic markers LC-3B and Beclin-1 in NRK-52E cells. **d** NRK-52E cells were treated as indicated and the expression of mammalian target of rapamycin (*mTOR*), phosphorylated mTOR, 4EBP-1, and P70S6K proteins was determined by Western blot. NRK-52E cells treated as mentioned for the in vitro experiment were divided into four groups: 1) control; 2) phosphate-buffered saline (*PBS*); 3) hucMSC-Ex; and 4) human fetal lung fibroblast-1exosomes (*HFL1-Ex*)

Due to autophagy playing significant roles in the homeostasis and physiological function of podocytes in the kidney [24], we detected the effect of hucMSC-Ex on activation of autophagy. NRK-52E cells were pre-treated with hucMSC-Ex or HFL1-Ex and then exposed to cisplatin, and autophagy-related indicators were tested. The results of transmission electron microscopy showed that there were a large number of autophagosomes in hucMSC-Ex-treated cells compared with the PBS and HFL1-Ex groups (Fig. 3a). To better illustrate the autophagy puncta, we used structured illumination microscopy (SIM) which allows us to acquire super-resolution two-dimensional images. mRFP-GFP-LC3B NRK-52E cells were pre-treated with hucMSC-Ex or HFL1-Ex and then exposed to cisplatin; SIM images showed that there are a large number of yellow and red dots in hucMSC-Ex-treated cells compared with the PBS and HFL1-Ex groups (Fig. 3b). hucMSC-Ex also increased the expression of autophagy-related genes including ATG5 and ATG7 by quantitative RT-PCR (see Additional file 3: Figure S3A and B) (*P* < 0.05). The quantitative results of the integrated density of the Western blot image showed that pre-treatment with hucMSC-Ex increased the expression of autophagic maker proteins such as LC3B and Beclin-1 (Fig. 3c). The mammalian target of rapamycin (mTOR) signaling pathway plays a critical role in negatively regulating autophagy. We further demonstrated that hucMSC-Ex inhibited the phosphorylation of mTOR and changed the expression of its downstream targets such as 4E-BP1 and P70S6K. The expression of 4E-BP1 was raised, and the expression of P70S6K reduced after hucMSC-Ex administration (Fig. 3d). In summary, these results indicate that pre-treatment with hucMSC-Ex inhibits mTOR phosphorylation and activates autophagy in renal tubule epithelial cells in response to cisplatin.

### hucMSC-Ex prevents cisplatin-induced apoptosis and secretion of inflammatory cytokines by activating autophagy in vitro

**Fig. 4:**
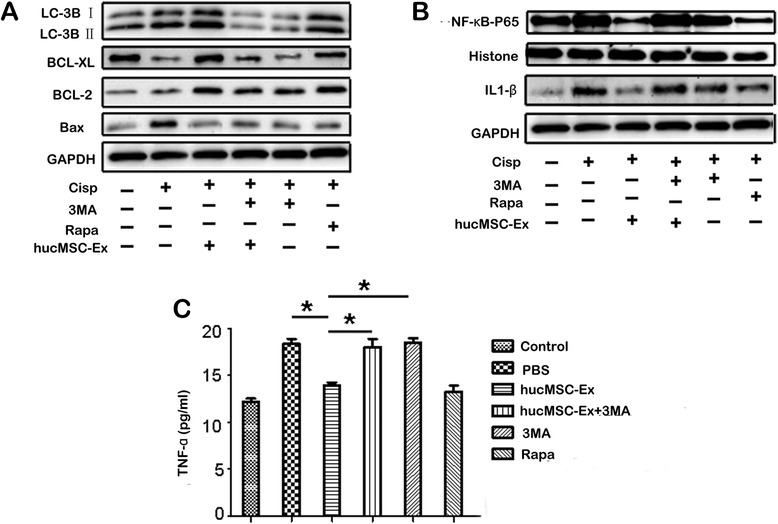
Human umbilical cord-derived mesenchymal stem cell exosomes (*hucMSC-Ex*) prevent cisplatin-induced apoptosis and the inflammatory response by activating autophagy in vitro. **a** The expression of LC3B, BCL-XL, BCL-2, Bax, and GAPDH proteins was measured by Western blot. **b** The expression of NF-κB-P65, histone, IL1-β, and GAPDH proteins was measured by Western blot. **c** ELISA analyses of tumor necrosis factor alpha (*TNF-α*) expression in NRK-52E cells. *P < 0.05, *n* = 3. NRK-52E cells treated as mentioned for the in vitro experiments were divided into six groups as follows: 1) control; 2) phosphate-buffered saline (*PBS*); 3) hucMSC-Ex; 4) hucMSC-Ex + 3-methyladenine (*3MA*); 5) 3MA; and 6) rapamycin (*Rapa*)

Considering the critical role of autophagy in cell apoptosis and tissue inflammation [25, 26], we wanted to know whether the preventative effects of hucMSC-Ex on cisplatin-induced apoptosis and inflammatory response are dependent on autophagy. We treated NRK-52E cells with hucMSC-Ex in the presence or absence of the autophagic inhibitor 3-methyladenine (3MA). As shown in Fig. 4a, 3MA inhibited the hucMSC-Ex-mediated increase of the autophagic marker protein LC3B and anti-apoptotic proteins BCL-XL and BCL-2, while it reversed the decrease of the pro-apoptotic protein Bax. However, treatment with the autophagic inducer rapamycin mimicked the effects of hucMSC-Ex, with an increase in LC3B, BCL-2, and BCL-XL proteins, and a decrease in Bax protein. We further wanted to know whether the effect of hucMSC-Ex on inflammatory cytokines is also influenced by an autophagic inhibitor. As shown in Fig. 4b, simultaneous treatment with 3MA abrogated the inhibitory role of hucMSC-Ex in cisplatin-induced IL-1β expression and NF-κB-P65 activation. Treatment with rapamycin reduced IL-1β expression and inactivated NF-κB-P65 to that observed in hucMSC-Ex-treated cells. The results of ELISA showed that, compared to the PBS group, TNF-α levels decreased in the hucMSC-Ex group but were restored in the hucMSC-Ex + 3MA group (Fig. 4c) (*P* < 0.05). Taken together, these results demonstrated that the activation of autophagy by hucMSC-Ex may contribute to its preventive effects on cisplatin-induced apoptosis and the inflammatory response in vitro.

### hucMSC-Ex-mediated activation of autophagy is required for the prevention of cisplatin-induced AKI in vivo

**Fig. 5:**
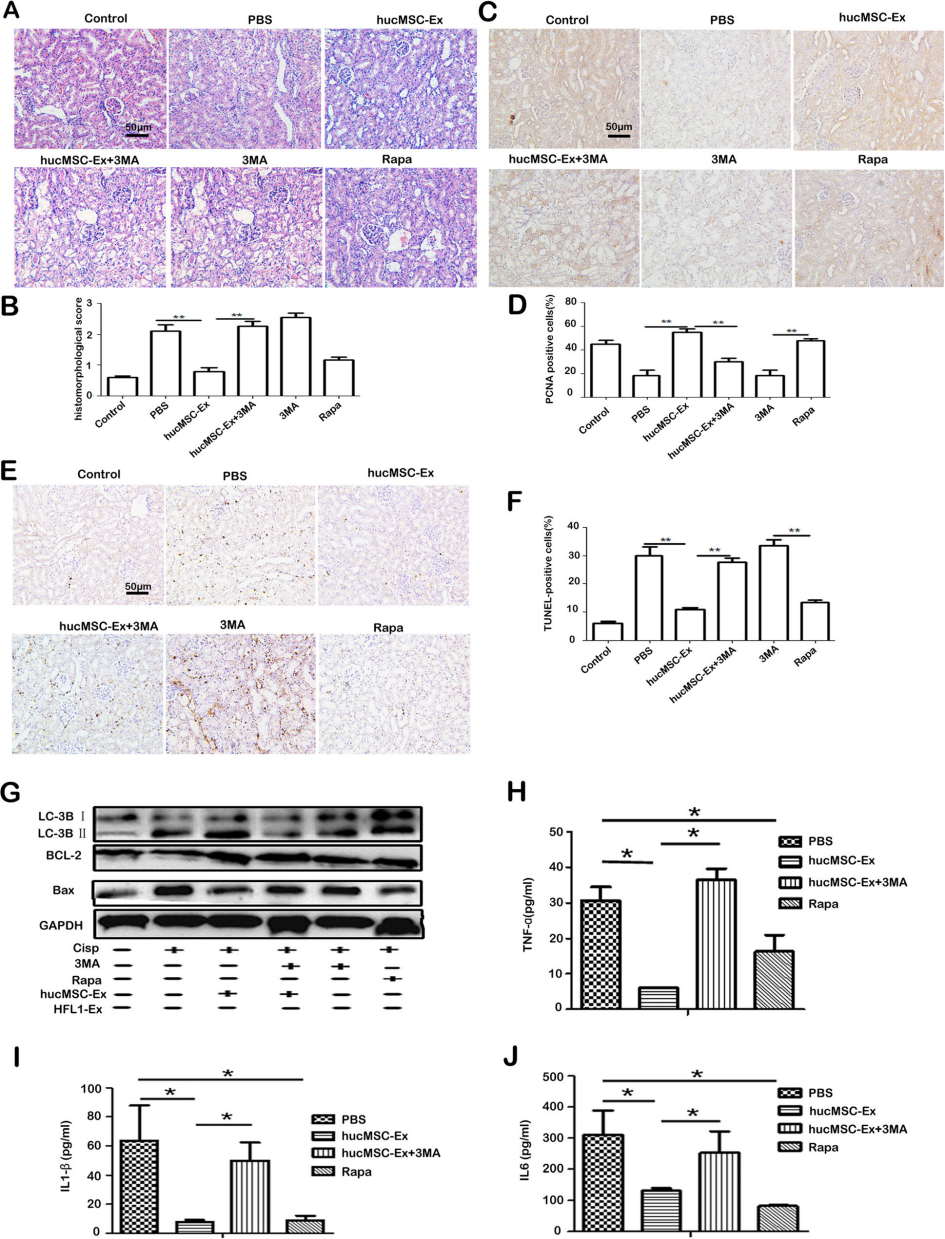
Human umbilical cord-derived mesenchymal stem cell exosomes (*hucMSC-Ex*) prevent apoptosis and secretion of inflammatory cytokines by activating autophagy in vivo. **a** Representative images of renal histology (200×, *scale bar* = 50 μm). **b** The histomorphological score. **c** Immunohistochemical analysis of PCNA expression in kidney tissues (200×, *scale bar* = 50 μm). **d** Percentage of proliferating cell nuclear antigen (*PCNA*)-positive cells. **e** TUNEL assay for apoptotic cells in rat renal tissues (200×, *scale bar* = 50 μm). **f** Percentage of apoptotic cells. **g** The expression of LC3B, BCL-2, Bax, IL1-β, and GAPDH proteins in the cisplatin-induced rat model were measured by Western blot. The serum inflammatory cytokines (**h**) tumor necrosis factor alpha (*TNF-α*), (**i**) interleukin-1β (*IL1-β*), and (**j**) interleukin-6 (*IL6*) were measured via Luminex assay. Data are expressed as mean ± SD. *P < 0.05, *n* = 6. Rats were divided into six groups as follows: 1) control; 2) phosphate-buffered saline (*PBS*); 3) hucMSC-Ex; 4) hucMSC-Ex + 3-methyladenine (*3MA*); 5) 3MA; and 6) rapamycin (*Rapa*)

In order to verify the activation of autophagy induced by the hucMSC-Ex in vivo model, 3MA (500 μg/kidney) and rapamycin (20 μg/kidney) were respectively injected under the kidney capsule before treatment with cisplatin. Representative images of H&E staining showed that the number of renal tubules with edema and structural damage were significantly reduced in the hucMSC-Ex group. However, co-injection with 3MA inhibited the protective effect of hucMSC-Ex on the renal structure. Rapamycin had a similar effect in preventing the structural damage of renal tubules to that of hucMSC-Ex (Fig. 5a and b). Furthermore, the number of PCNA-positive cells increased in the hucMSC-Ex group compared to the PBS group. 3MA inhibited the effect of hucMSC-Ex. The expression of PCNA slightly increased in the rapamycin group (Fig. 5c and d). As shown in Fig. 5e and f, the number of TUNEL-positive cells in the hucMSC-Ex and rapamycin groups was reduced, but showed few changes in the hucMSC-Ex + 3MA and HFL1-Ex groups compared to that in the PBS group. The results of immunohistochemical staining showed that TNF-α levels were significantly lower in the hucMSC-Ex and rapamycin groups than in the PBS group. However, TNF-α expression in the hucMSC-Ex + 3MA and 3MA groups showed no significant difference to that in the PBS group (see Additional file 4: Figure S4). Western blotting analyses showed that the expression of the autophagic marker protein LC3B and anti-apoptotic protein BCL-2 increased, while the expression of the pro-apoptotic protein Bax decreased in the hucMSC-Ex group (Fig. 5g). However, the effects of hucMSC-Ex on these proteins were inhibited by 3MA. Luminex assays showed that the serum levels of the inflammatory cytokines TNF-α, IL1-β, and IL6 were significantly lower in the hucMSC-Ex and rapamycin groups than in the PBS group. However, their expression in the hucMSC-Ex + 3MA group showed no significant difference to that in the PBS group (Fig. 5h–j). Consequently, these results indicate that the activation of autophagy is involved in the preventative role of hucMSC-Ex in cisplatin-induced AKI.

## Discussion

Increasing evidence suggests that the administration of exogenous mesenchymal stem cells (MSCs) improves the recovery of injured tissue, including acute kidney injury (AKI) [10–12, 27–31]. Several studies have demonstrated that the administration of MSCs could reverse kidney injury through paracrine mechanisms rather than by MSC transdifferentiation [32, 33]. MSC exosomes might be such a paracrine mechanism for cell-to-cell communication. Exosomes are small vesicles released by cells bearing the surface antigens characteristic of the cell of origin [34, 35]. These vesicles may play critical roles in cell communication by transferring RNA, proteins, and bioactive lipids [36]. Exosomes may be more advantageous than stem cells in regenerative medicine due to the avoidance of possible long-term pathologic differentiation of engrafted cells or tumor generation [37, 38]. The release of exosomes may be constitutive, or a consequence of cell activation by soluble agonists, or physical and chemical stresses such as oxidative stress and hypoxia, or by shear stress [39]. We demonstrated in this study that pre-treatment with hucMSC-Ex could inhibit cisplatin-induced apoptosis and the secretion of inflammatory cytokines in renal proximal tubule epithelial cells. hucMSC-Ex pretreatment suppressed the increase of BUN and serum Cr levels, as well as the deterioration of proximal tubule epithelial cells induced by cisplatin.

The role of autophagy as a degradative pathway is critical in regenerative medicine. Many reports show that basal or physiological autophagy contributes to the maintenance of cellular homeostasis and quality control of proteins and subcellular organelles. Under pathological conditions or cell stress, autophagy is induced which may serve as an adaptive and protective mechanism for cell survival. Autophagy is essential to the homeostasis and physiological function of podocytes in the kidney [40]. Notably, autophagy induction as a self-protection mechanism has been demonstrated in renal tubular cells in experimental models of AKI caused by ischemia-reperfusion and nephrotoxicants such as cisplatin and cyclosporine [41–44]. Recent studies also report that autophagy play an important role in MSC-promoted tissue regeneration [45, 46]. Baixauli et al. [47] note that the emerging function of exosomes as a means of alleviating intracellular stress conditions in coordination with the autophagy-lysosomal pathway is essential for preserving intracellular protein and RNA homeostasis. However, whether hucMSC-Ex can activate target cell autophagy in advance to prevent tissue injury is not reported. Here, we found that hucMSC-derived exosomes can induce target cell autophagy to prevent nephrotoxicity in the early injury stage. In our study, we found that hucMSC-Ex upregulated the expression of the autophagic marker protein LC3B through the inhibition of mTOR phosphorylation. mTOR is an evolutionarily conserved nutrient-sensing serine/threonine protein kinase, with a critical role in regulating protein synthesis and autophagy [48, 49]. mTOR signaling negatively regulates autophagy, and mTOR suppression by rapamycin contributes to the induction of autophagy [50]. We found that hucMSC-Ex downregulated the expression of phosphorylated mTOR and changed the levels of expression of p70S6K and 4EBP1, suggesting that hucMSC-Ex may activate autophagy by inhibiting the mTOR signaling pathway.

In order to prove whether hucMSC-Ex-mediated autophagy is essential for the inhibition of apoptosis and secretion of inflammatory cytokines, we pre-treated NRK-52E cells with the specific inhibitor of autophagy, 3-methyladenine (3MA). Inhibition of autophagy reduces the effects of hucMSC-Ex on anti-apoptosis and the inhibition of inflammatory cytokines. On the other hand, the autophagic inducer rapamycin has a similar effect on renal protection to that of hucMSC-Ex both in vitro and in vivo, suggesting that induction of autophagy is essential for the protective role of hucMSC-Ex in cisplatin-induced renal injury.

**Fig. 6:**
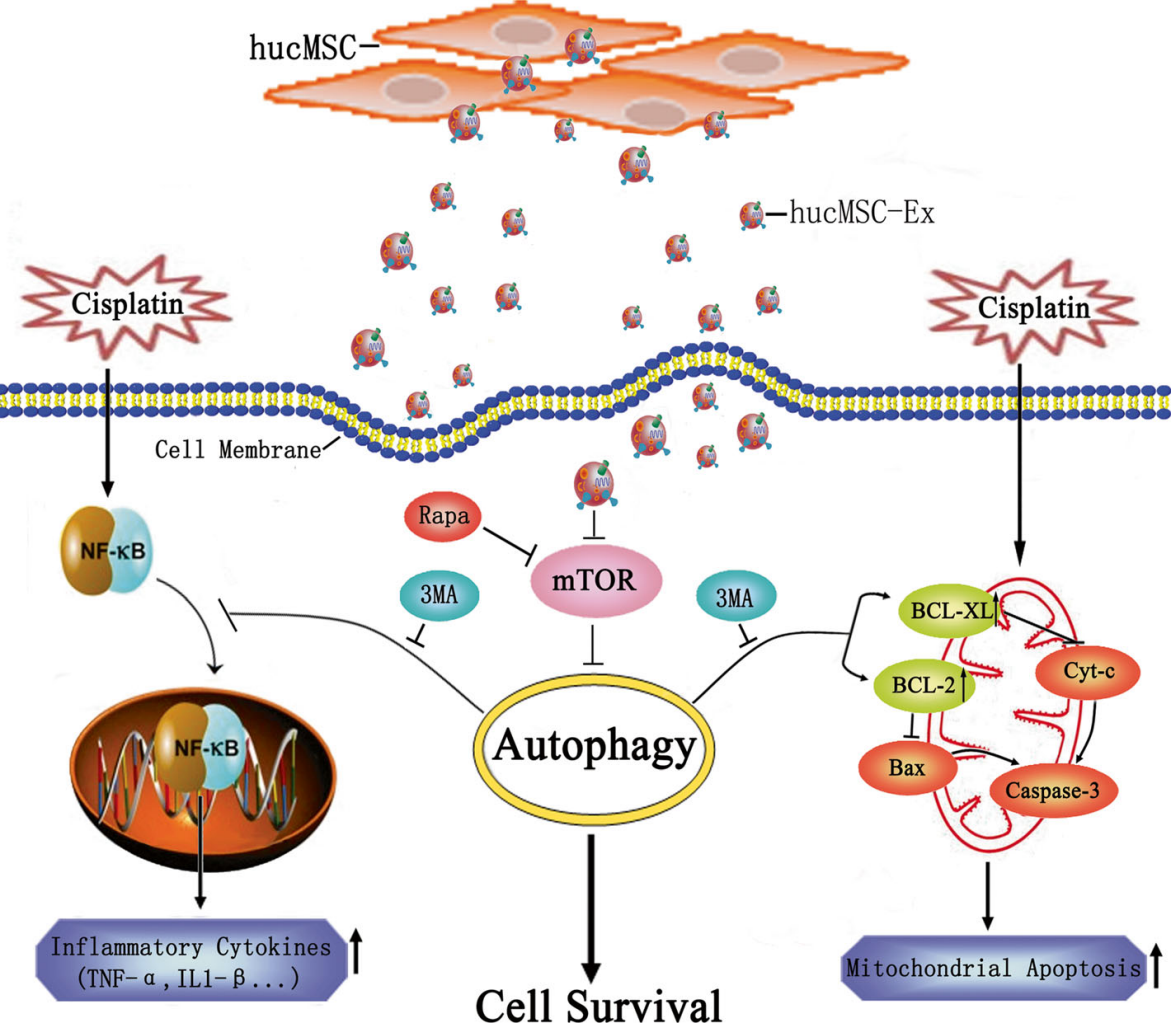
A proposed model for the mechanism of human umbilical cord-derived mesenchymal stem cell exosome (*hucMSC-Ex*)-mediated protection against cisplatin-induced AKI. hucMSC-Ex activates autophagy and leads to the reduction in cell apoptosis and inflammatory responses, and the improvement of renal recovery. *3MA* 3-methyladenine, *IL* interleukin, *mTOR* mammalian target of rapamycin, *Rapa* rapamycin, *TNF* tumor necrosis factor

From the above discussion, we propose a model as delineated in Fig. 6. Cisplatin induces apoptosis and inflammation in renal proximal tubule epithelial cells, resulting in acute renal injury. hucMSC-Ex pretreatment activates autophagy through the inhibition of the mTOR signaling pathway in renal proximal tubule epithelial cells, leading to the reduction of cell apoptosis and inflammatory responses and the improvement in renal recovery. The preventive effects of hucMSC-Ex on cisplatin-induced AKI can be reversed by the autophagic inhibitor 3-MA. Inactivation of mTOR by rapamycin mimics the effects of hucMSC-Ex on cisplatin-induced AKI.

## Conclusion

This study reveals that hucMSC-derived exosomes prevent against cisplatin-induced AKI through an autophagy-related mechanism. These findings provide a basis for the future use of exosomes as a new biological therapeutic approach for renal diseases and injuries.

## Additional files

Additional file 1: Figure S1. Characterization of hucMSCs. (A) Immunophenotyping of hucMSC. Histograms of MSCs stained with anti-CD13, CD44, CD29, CD90, CD105, HLA-1, CD45, HLA-DR, and CD34 shown with an overlaid isotype control. (B) Multi-lineage differentiation of hucMSCs. Adipogenic differentiation was assayed by Oil Red O staining (100×, *scale bar* = 50 μm). (C) Osteogenic differentiation was assayed by alkaline phosphatase staining (100×, *scale bar* = 50 μm). (PDF 270 kb)

Additional file 2: Figure S2. Characterization of hucMSC-derived exosomes. (A) Nanoparticle tracking analysis on characteristics of the particles of hucMSC-Ex: (a) particle size and concentration, (b) the relative-intensity three-dimensional plot, (c) substantial shape of exosomes. (B) The morphology of hucMSC-Ex was observed under transmission electron microscopy (*scale bar* = 100 nm). (C) Western blotting analyses of the expression of exosomal markers CD81, CD9, and CD63 in hucMSC-Ex. (D) TUNEL assay was performed to detect the apoptotic cells (200×, *scale bar* = 50 μm). (E) Electron microscope analyses of the expression of exosomal markers CD9 colloidal gold in hucMSC-Ex (*scale bar* = 500 nm). (PDF 228 kb)

Additional file 3: Figure S3. hucMSC-Ex can activate autophagy in vitro. Quantitative RT-PCR analyses of ATG5 (A) and ATG7 (B) mRNA levels in NRK-52E cells. *P < 0.05, *n* = 3. (PDF 83 kb)

Additional file 4: Figure S4. hucMSC-Ex prevents secretion of inflammatory cytokines by activating autophagy in vivo. (A) Immunohistochemical analysis of TNF-α in kidney tissues (100×, *scale bar* = 100 μm). (PDF 197 kb)

## Abbreviations

3MA: 3-methyladenine
AKI: Acute kidney injury
BUN: Blood urea nitrogen
Cr: Creatinine
ELISA: Enzyme-linked immunosorbent assay
FACS: Fluorescence-activated cell sorting
FITC: Fluorescein isothiocyanate
HFL1: Human fetal lung fibroblast-1
hucMSC: Human umbilical cord-derived mesenchymal stem cell
hucMSC-Ex: Human umbilical cord-derived mesenchymal stem cell exosomes
IL: Interleukin
LG-DMEM: Low-glucose Dulbecco’s modified Eagle’s medium
MEM: Minimum essential medium
MSC: Mesenchymal stem cell
mTOR: Mammalian target of rapamycin
MV: Microvesicle
NTA: Nanoparticle tracking analysis
PBS: Phosphate-buffered saline
PE: Phycoerythrin
qRT-PCR: Quantitative real-time polymerase chain reactiuon
TNF: Tumor necrosis factor
TUNEL: Terminal deoxynucleotidyl transferase-mediated dUTP nick end-labeling
