# Supplementary material for: Pre-incubation with hucMSC-exosomes prevents cisplatin-induced nephrotoxicity by activating autophagy
Source: Stem Cell Res Ther. 2017 Apr 8;8:75. doi: 10.1186/s13287-016-0463-4 (PMC5385032; doi:10.1186/s13287-016-0463-4)
Supplement: Supplementary file 1 — Characterization of hucMSCs. (A) Immunophenotyping of hucMSC. Histograms of MSCs stained with anti-CD13, CD44, CD29, CD90, CD105, HLA-1, CD45, HLA-DR, and CD34 shown with an overlaid isotype control. (B) Multi-lineage differentiation of hucMSCs. Adipogenic differentiation was assayed by Oil Red O staining (100×, scale bar = 50 μm). (C) Osteogenic differentiation was assayed by alkaline phosphatase staining (100×, scale bar = 50 μm). (PDF 270 kb) [file 13287_2016_463_MOESM1_ESM.pdf]

Additional file 1

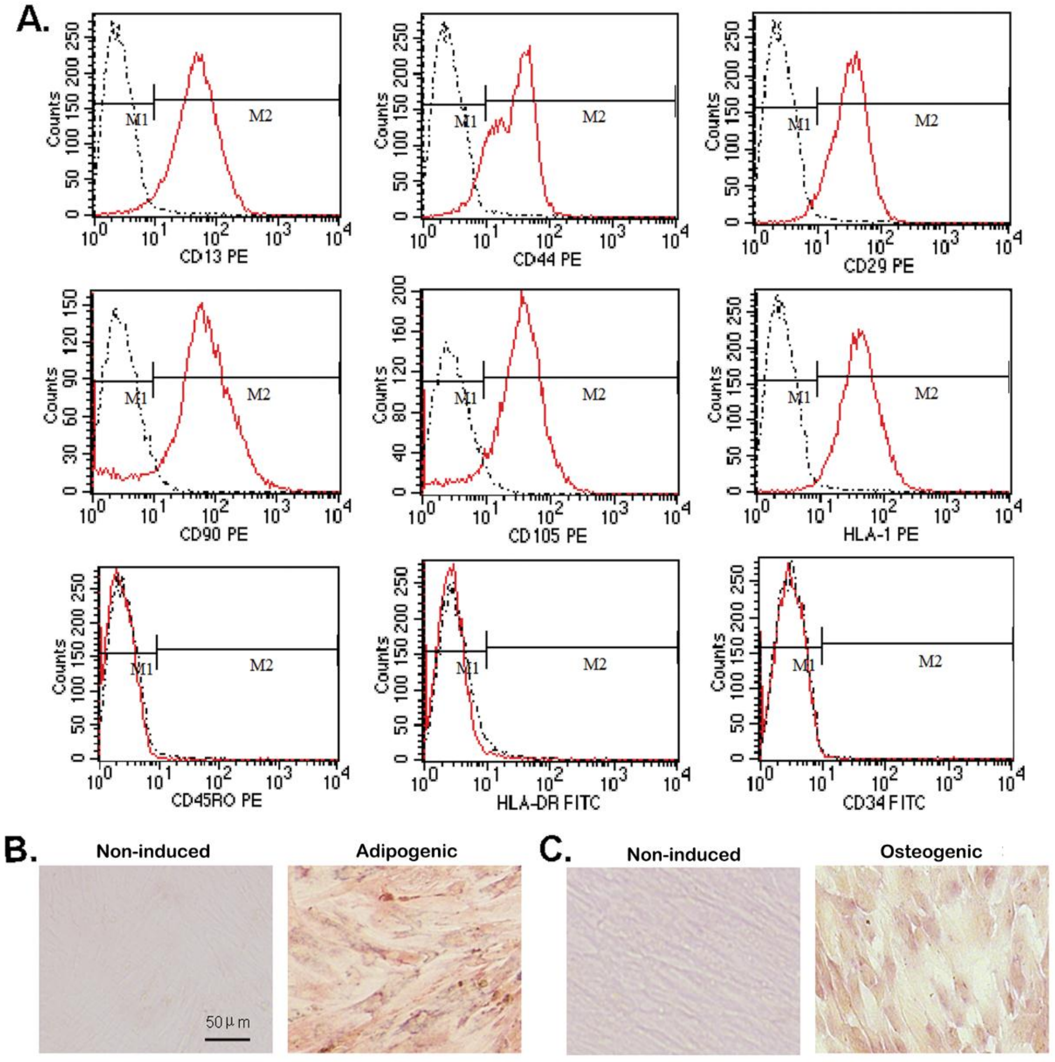

Figure. S1. Characterization of hucMSCs. (A) Immunophenotyping of hucMSC. Histograms of MSCs stained with anti-CD13, CD44, CD29, CD90, CD105, HLA-1, CD45, HLA-DR and CD34 shown with an overlaid isotype control. (B) Multi-lineage differentiation of hucMSC. Adipogenic differentiation was assayed by Oil Red O staining (100×, bar=50μm). (C) Osteogenic differentiation was assayed by alkaline phosphatase staining (100×, bar=50μm).
