# Supplementary material for: Pre-incubation with hucMSC-exosomes prevents cisplatin-induced nephrotoxicity by activating autophagy
Source: Stem Cell Res Ther. 2017 Apr 8;8:75. doi: 10.1186/s13287-016-0463-4 (PMC5385032; doi:10.1186/s13287-016-0463-4)
Supplement: Supplementary file 2 — Characterization of hucMSC-derived exosomes. (A) Nanoparticle tracking analysis on characteristics of the particles of hucMSC-Ex: (a) particle size and concentration, (b) the relative-intensity three-dimensional plot, (c) substantial shape of exosomes. (B) The morphology of hucMSC-Ex was observed under transmission electron microscopy (scale bar = 100 nm). (C) Western blotting analyses of the expression of exosomal markers CD81, CD9, and CD63 in hucMSC-Ex. (D) TUNEL assay was performed to detect the apoptotic cells (200×, scale bar = 50 μm). (E) Electron microscope analyses of the expression of exosomal markers CD9 colloidal gold in hucMSC-Ex (scale bar = 500 nm). (PDF 228 kb) [file 13287_2016_463_MOESM2_ESM.pdf]

Additional file 2

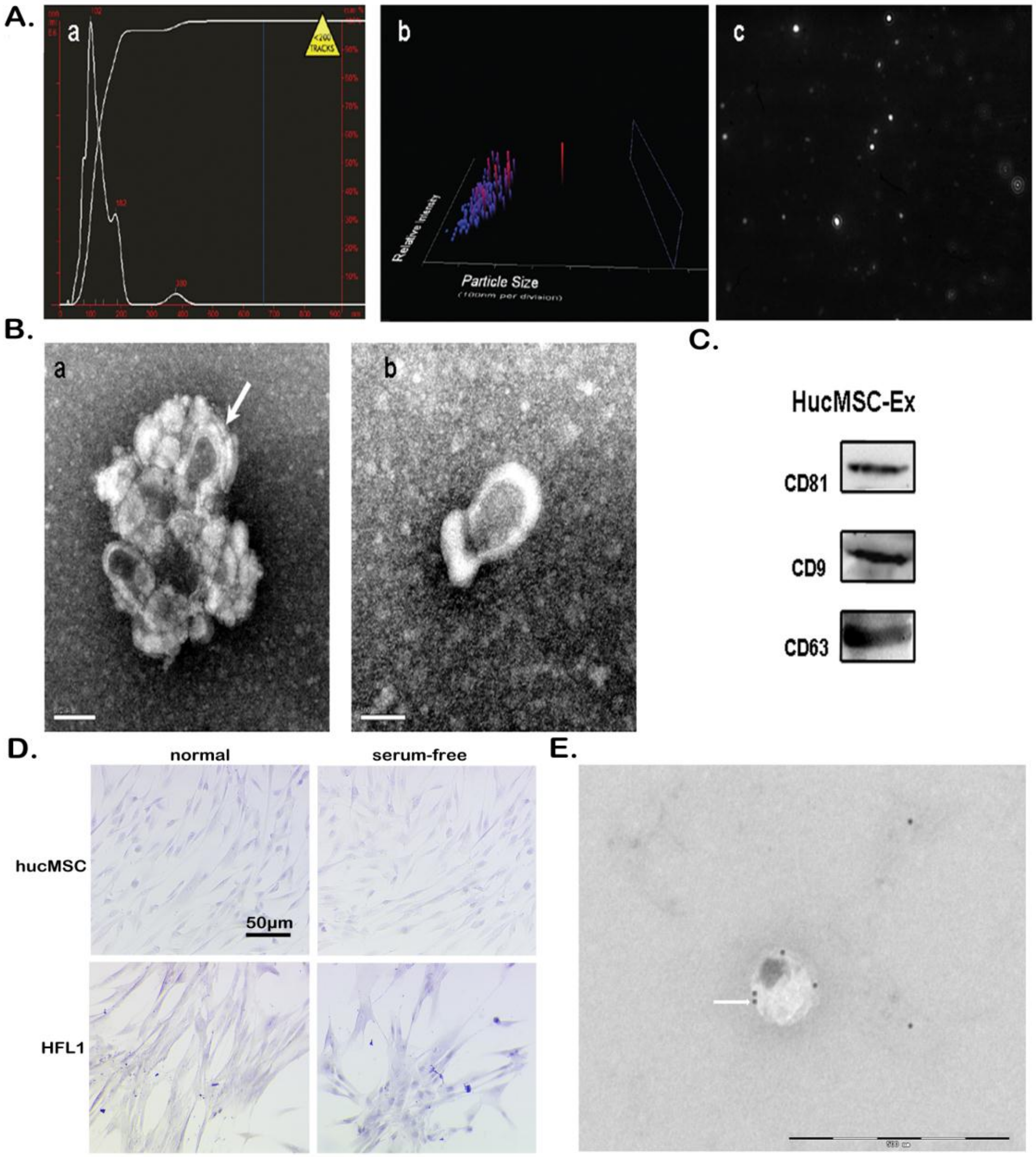

Figure. S2. Characterization of hucMSC derived exosomes.(A) Nanoparticle tracking analysis on characteristics of the particles of hucMSC-Ex. (a. particle size and concentration, b. the relative intensity 3D plot, c. substantial shape of exosomes). (B) The morphology of hucMSC-Ex was observed under transmission electron microscopy (bar=100 nm). (C) Western blotting analyses of the expression of exosomal markers CD81, CD9 and CD63 in hucMSC-Ex. (D) TUNEL assay was performed to detect the apoptotic cells (200×, bar=50μm). (E) Electron microscope analyses of the expression of exosomal markers CD9 colloidal gold in hucMSC-Ex (bar=500nm).
