# Supplementary material for: Pre-incubation with hucMSC-exosomes prevents cisplatin-induced nephrotoxicity by activating autophagy
Source: Stem Cell Res Ther. 2017 Apr 8;8:75. doi: 10.1186/s13287-016-0463-4 (PMC5385032; doi:10.1186/s13287-016-0463-4)
Supplement: Supplementary file 3 — hucMSC-Ex can activate autophagy in vitro. Quantitative RT-PCR analyses of ATG5 (A) and ATG7 (B) mRNA levels in NRK-52E cells. *P < 0.05, n = 3. (PDF 83 kb) [file 13287_2016_463_MOESM3_ESM.pdf]

Additional file 3

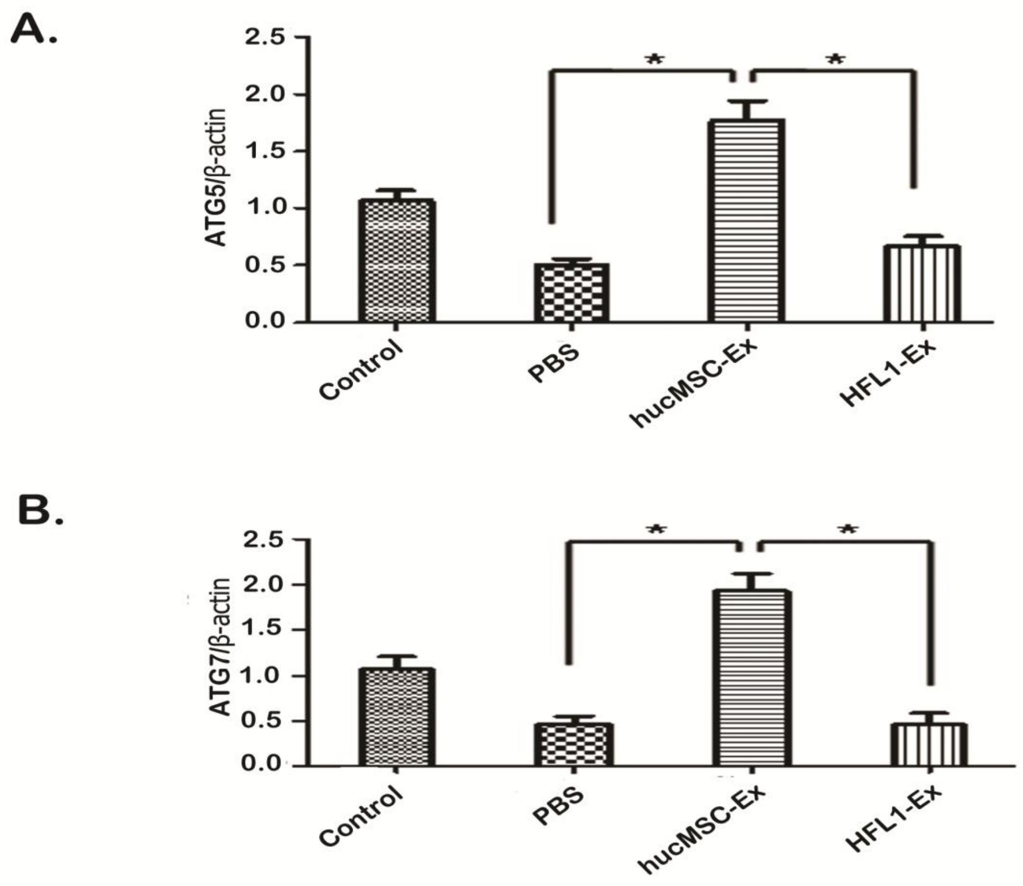

Figure. S3. HucMSC-Ex can activate autophagy in vitro. (A), (B) Quantitative RT-PCR analyses of ATG5 (A) and ATG7 (B) mRNA levels in NRK-52E cells (*P<.05, n=3).
