# Supplementary material for: Pre-incubation with hucMSC-exosomes prevents cisplatin-induced nephrotoxicity by activating autophagy
Source: Stem Cell Res Ther. 2017 Apr 8;8:75. doi: 10.1186/s13287-016-0463-4 (PMC5385032; doi:10.1186/s13287-016-0463-4)
Supplement: Supplementary file 4 — hucMSC-Ex prevents secretion of inflammatory cytokines by activating autophagy in vivo. (A) Immunohistochemical analysis of TNF-α in kidney tissues (100×, scale bar = 100 μm). (PDF 197 kb) [file 13287_2016_463_MOESM4_ESM.pdf]

Additional file 4

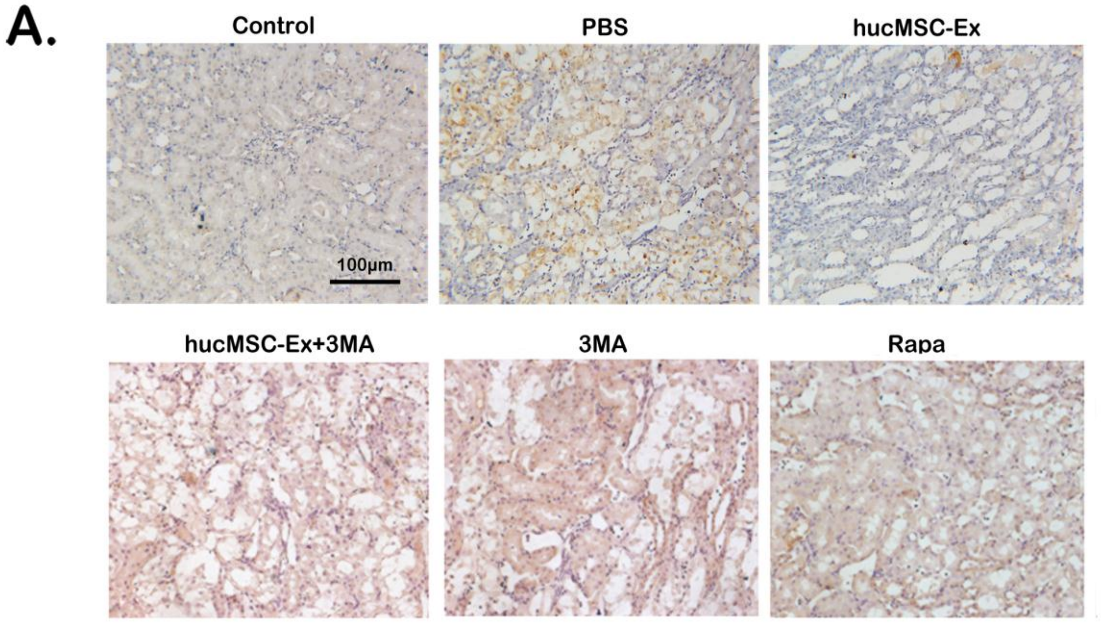

Figure. S4. HucMSC-Ex prevents secretion of inflammation by activating autophagy in vivo. (A) Immunohistochemical analysis of TNF-α in kidney tissues (100×, bar=100μm).
